# Bottom-up modeling approach for the quantitative estimation of parameters in pathogen-host interactions

**DOI:** 10.3389/fmicb.2015.00608

**Published:** 2015-06-19

**Authors:** Teresa Lehnert, Sandra Timme, Johannes Pollmächer, Kerstin Hünniger, Oliver Kurzai, Marc Thilo Figge

**Affiliations:** ^1^Applied Systems Biology, Leibniz Institute for Natural Product Research and Infection Biology - Hans-Knöll-InstituteJena, Germany; ^2^Faculty of Biology and Pharmacy, Friedrich Schiller University JenaJena, Germany; ^3^Fungal Septomics, Septomics Research Center, Friedrich Schiller University and Leibniz Institute for Natural Product Research and Infection Biology Hans-Knöll-InstituteJena, Germany

**Keywords:** state-based model, agent-based model, pathogen-host interaction, parameter estimation, whole-blood infection assay, *Candida albicans*

## Abstract

Opportunistic fungal pathogens can cause bloodstream infection and severe sepsis upon entering the blood stream of the host. The early immune response in human blood comprises the elimination of pathogens by antimicrobial peptides and innate immune cells, such as neutrophils or monocytes. Mathematical modeling is a predictive method to examine these complex processes and to quantify the dynamics of pathogen-host interactions. Since model parameters are often not directly accessible from experiment, their estimation is required by calibrating model predictions with experimental data. Depending on the complexity of the mathematical model, parameter estimation can be associated with excessively high computational costs in terms of run time and memory. We apply a strategy for reliable parameter estimation where different modeling approaches with increasing complexity are used that build on one another. This bottom-up modeling approach is applied to an experimental human whole-blood infection assay for *Candida albicans*. Aiming for the quantification of the relative impact of different routes of the immune response against this human-pathogenic fungus, we start from a non-spatial state-based model (SBM), because this level of model complexity allows estimating *a priori* unknown transition rates between various system states by the global optimization method *simulated annealing*. Building on the non-spatial SBM, an agent-based model (ABM) is implemented that incorporates the migration of interacting cells in three-dimensional space. The ABM takes advantage of estimated parameters from the non-spatial SBM, leading to a decreased dimensionality of the parameter space. This space can be scanned using a local optimization approach, i.e., *least-squares error estimation* based on an *adaptive regular grid search*, to predict cell migration parameters that are not accessible in experiment. In the future, spatio-temporal simulations of whole-blood samples may enable timely stratification of sepsis patients by distinguishing hyper-inflammatory from paralytic phases in immune dysregulation.

## 1. Introduction

The human fungal pathogen *Candida albicans* is part of the normal microbial flora in more than half of the global population. In immunocompromised patients it can become invasive and may enter the blood stream via medical devices, e.g., catheters, or translocation in the gut and can cause severe systemic infections. The immune response against *C. albicans* in human blood comprises the interplay of various complex biological processes involving different immune mechanisms (Duggan et al., 2015b). Most importantly, the whole-blood infection assay allows multiple immune effector mechanisms to occur at the same time and thus modulate the overall outcome (Luo et al., 2013; Cunha et al., 2014; Hünniger et al., 2015). Applying a systems biology approach, we quantified individual processes and in this way revealed the main route of the immune response against *C. albicans* in human blood (Hünniger et al., 2014). This was achieved by an iterative systems biology cycle involving experiment, mathematical modeling, hypothesis generation and further experimental investigation.

The choice of an appropriate mathematical modeling approach strongly depends on the questions to be answered and the hypothesis, as well as the characteristics of the underlying experimental data with regard to temporal and spatial information. A wide range of modeling approaches exists that differ by their computational complexity and can be classified depending on the degree of spatial representation as well as the internal degrees of freedom attributed to the model entities. The computationally cheapest modeling approach for dynamic systems is represented by ordinary differential equations (ODE), where biological entities are assumed to be present in high numbers and spatial information is not required such that they can be collectively represented by a homogeneously distributed concentration variable. State-based models (SBM) resolve the biological entities as individuals that occupy states and are able to perform transitions between states representing dynamic processes. In contrast to ODE, this approach allows modeling discrete events for any entity number in a biological system. However, SBM are in turn limited in that they do not represent spatial aspects. Individual-based models (IBM) such as cellular automata (CA) and agent-based models (ABM) do simulate discrete entities in space and time (Medyukhina et al., 2015). In a CA simulation, these entities can undergo state changes associated with their internal degrees of freedom as well as positional changes on a pre-defined spatial grid of computational cells (Von Neumann, 1951; Bittig and Uhrmacher, 2010). The discrete number of individual entities as well as the spatial representation of the environment result in increasing computational costs in terms of run-time and memory. Even more computationally expensive but biologically more realistic simulations can be performed by the ABM approach. Here, biological objects are represented as individual entities, so-called agents, that are able to move in space and can act as well as interact with other agents according to individual properties. Examples of ABM for the pathogen-host interaction between the human-pathogenic fungus *Aspergillus fumigatus* and phagocytes were presented by Tokarski et al. (2012) and Pollmächer and Figge (2014). In particular, the ABM developed by Pollmächer and Figge (2014) simulates the detection of *A. fumigatus* conidia by macrophages in a to-scale representation of human alveoli and predicts the requirement of a chemotactic signal guiding the phagocytes to the spatial positions of conidia.

In general, parameters of bio-mathematical models characterize the components by their morphology and their dynamic behavior. For example, cells may be defined by parameters for size and shape as well as by parameters for interactions in the spatial environment that are associated with the typical frequency of interaction processes. Model parameters associated with dynamical, functional and morphological aspects of biological processes may be extracted from microscopic images by applying an image-based systems biology approach (Horn et al., 2012; Mech et al., 2014; Medyukhina et al., 2015). However, in many cases microscopy experiments cannot be performed for technical reasons, as is also the case for whole-blood infection assays where the majority of cells are erythrocytes blocking the view on leukocytes, let alone fungal pathogens that are present in even lower numbers. In situations like these, numerical estimation of *a priori* unknown parameter values by comparison with experimental time-series data becomes a highly relevant issue. Parameter estimation algorithms are applied to find the optimal match between the experimental data and simulated model data. These optimization algorithms can be characterized by their search technique within the parameter space, i.e., as global or local approaches, and their mathematical procedures, i.e., as stochastic or deterministic approaches (Moles et al., 2003; Ashyraliyev et al., 2009). Local optimization techniques search for better parameter values within a locally restricted parameter space, where the direct search method and gradient based methods are widely used (Ashyraliyev et al., 2009). They show fast convergence to the optimal parameter values, but since local optimization algorithms will get stuck in a nearby local optimum, an educated guess of the initial parameter values is absolutely required. In contrast, global optimization strategies search a wide range of the parameter space with possibly various local optima and the subclass of deterministic optimization strategies can find the global optimum with pre-defined accuracy (Ashyraliyev et al., 2009). High-dimensional parameter spaces may be searched by stochastic optimization algorithms that make use of probabilistic elements to avoid getting trapped in local optima in order to find the global optimum. Common stochastic search algorithms of this type are Metropolis Monte Carlo (MMC) (Metropolis et al., 1953), adaptive random search and evolutionary computation techniques such as differential evolution (DE) (Storn and Price, 1997). Additionally, heuristics can be applied in support of a fast convergence rate of global or local optimization strategies, e.g., simulated annealing (SA) (Kirkpatrick et al., 1983; Gonzalez et al., 2007), great deluge (Dueck, 1993), or performing multiple searches from random start parameters. The selection of the most suitable optimization algorithm depends on specific model properties, such as the dimension of the parameter space and the computational costs for the model simulations that have to be repeatedly performed. For computationally cheap ODE models, the computationally expensive stochastic global optimization algorithms may be used, such as DE applied by Hernandez-Vargas et al. (2014) and SA based on MMC applied by Hünniger et al. (2014) and Mech et al. (2014).

The non-spatial virtual infection model of the immune response against *C. albicans* in human blood was formulated as a SBM and its parameters were fitted to the experimentally determined time-evolution of concentrations for *C. albicans* cells that are alive or killed and that can either reside in extracellular space or inside immune cells of different types, i.e., monocytes or granulocytes (polymorphonuclear neutrophils, PMN) (Hünniger et al., 2014). Furthermore, we observed a cell population of *C. albicans* that remained alive or killed in extracellular space, i.e., these fungal cells are resistant against phagocytosis and/or killing. The different *C. albicans* cell populations were assigned states and individual cells could perform transitions between states, such as phagocytosis by immune cells, subsequent intracellular killing, extracellular killing by antimicrobial peptides or acquiring resistance against phagocytosis and/or killing. Resistant *C. albicans* cells are a population of cells that were found to be protected against phagocytosis and/or killing and that remained in the extracellular space of the whole-blood infection assay (Hünniger et al., 2014). Since the model is restricted to the dynamics of states occupied by pathogenic cells we refer to the model by Hünniger et al. (2014) as P-SBM. In the present study, motivated by newly measured experimental data regarding the immune cell number of monocytes and PMN in the whole-blood assays, we take the next step and modify the P-SBM to drop its implicit assumption that the number of immune cells for samples from different individuals would be the same. Since in the modified SBM states are assigned to the pathogenic cells as well as to the two types of immune cells, which have been found to actively participate in *C. albicans* elimination, we will refer to this model as PI-SBM. Taking individual immune cells explicitly into account obviously makes the simulations of the whole-blood infection assay more realistic, albeit at the expense of higher computational costs for global parameter optimization that will be performed using SA based on the MMC scheme as was the case for the P-SBM.

**Figure 1:**
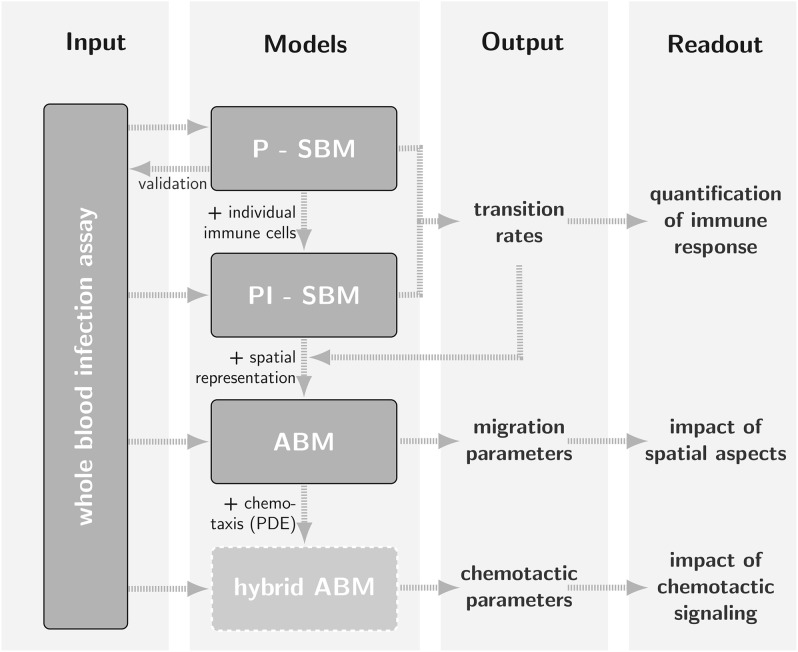
**Schematic representation of the bottom-up modeling approach as a strategy for parameter estimation where different mathematical models with increasing levels of complexity build on one another**. Using model outputs at one level for calibration of models at a higher level is by way of example demonstrated for *Candida albicans* infection in a human whole-blood assay (Hünniger et al., 2014). The state-based model P-SBM, which focuses on the viewpoint of pathogens, was modified into the state-based model PI-SBM by accounting for actions of individual immune cells. After calibration of transition rates in the non-spatial state-based model, these were used to simulate the infection process in an agent-based model (ABM) that accounts for the spatial representation of the whole-blood infection assay. In the future, the calibration of migration parameters may for example serve as input for a hybrid ABM that captures the time-evolution of chemotactic signaling by solving reaction-diffusion equations.

A timely stratification of sepsis patients in different phases of immune dysregulation requires spatio-temporal simulations of whole-blood samples. To achieve this goal, an ABM of the whole-blood infection assay was established that builds on the PI-SBM and incorporates spatial properties of the blood sample in a three-dimensional continuous representation. In particular, in the ABM *C. albicans* cells as well as monocytes and PMN are agents that can migrate in the environment and interact with each other. Apart from the model parameters associated with the migration of cells, the ABM was based on the transition rates of the PI-SBM after appropriate conversion. This procedure strongly reduces the number of *a priori* unknown parameters of agents to the subset of migration parameters. The latter can be estimated using the computationally cheap grid search algorithm and enables the prediction of the migration behavior for the different immune cell types that are otherwise not directly accessible in experiment. The interrelations between the different modeling approaches are schematically shown in Figure 1 demonstrating that results are re-used across different modeling approaches to simultaneously facilitate an increase in model complexity and a decrease in computational expense for parameter estimation. Our step-wise computational biology approach avoids typical limitations of realistic models by focusing parameter estimation on those parameters that arise at the next level of model complexity.

## 2. Materials and methods

### 2.1. Non-spatial state-based model

The initial version of the non-spatial SBM describes the dynamics of state transitions for the human-pathogenic fungus *C. albicans* in whole-blood samples of healthy donors (Hünniger et al., 2014). In agreement with experimental data, the time-evolution of different *C. albicans* cells that are alive or killed and in extracellular space or phagocytosed by either monocytes or PMN can be simulated in this way. Since this SBM assumes the number of immune cells to be constant across blood samples of different donors and does only simulate the dynamics of the pathogenic (P) cells, it is hereafter referred to as P-SBM. However, it is known that the number of immune cells may strongly vary across human individuals and in particular for patients. Therefore, we increase the model complexity by advancing the P-SBM to a model that does explicitly account for the number of immune cells being present in a hemogram. Data including immune cell counts can easily be obtained both in an experimental as well as in a clinical setting. This model is hereafter referred to as PI-SBM to indicate that state transitions are computed for pathogenic (P) as well as immune (I) cells.

For comparison between the model predictions and the experimentally determined kinetics in the whole-blood infection assay, we introduce specific combinations of states, referred to as *combined units*, that are measurable and useable for the parameter estimation. These comprise all extracellular *C. albicans* cells *C_E_*,

(1) $$C_{E}\equiv C_{AE}+C_{KE}+C_{AR}+C_{KR},$$

that are either alive (*C_AE_*) or killed (*C_KE_*) cells in extracellular space as well as cells resistant against killing and/or phagocytosis that are either alive (*C_AR_*) or killed (*C_KR_*). Next, the combined units *C_M_* and *C_G_* refer to *C. albicans* cells that are phagocytosed, respectively, by monocytes

(2) $$C_{M}\equiv \sum_{i\geq 0}\sum_{j\geq 0}M_{i,j}(i+j),$$

or by granulocytes

(3) $$C_{G}\equiv \sum_{i\geq 0}\sum_{j\geq 0}G_{i,j}(i+j).$$

Here, *M*_*i*, *j*_ and *G*_*i*, *j*_ refer to the number of monocytes and granulocytes (PMN), respectively, with *i* alive and *j* killed phagocytosed *C. albicans* cells. We limit the maximal number of *C. albicans* cells that can be phagocytosed by an immune cell to 18, i.e., *i*, *j* < 10, being much larger than observed in experiment (Hünniger et al., 2014). Furthermore, all killed *C. albicans* cells are given by the combined unit

(4) $$C_{K}\equiv C_{KE}+C_{KR}+\sum_{i\geq 0}\sum_{j\geq 1}(M_{i,j}+G_{i,j})j,$$

and all alive *C. albicans* cells by the combined unit

(5) $$C_{A}\equiv C_{AE}+C_{AR}+\sum_{i\geq 1}\sum_{j\geq 0}(M_{i,j}+G_{i,j})i.$$

It should be noted that only three of the five combined units are independent of each other, due to the conservation relations *C* = *C_E_* + *C_G_* + *C_M_* and *C* = *C_K_* +*C_A_* for the total number of *C. albicans* cells *C*.

**Figure 2:**
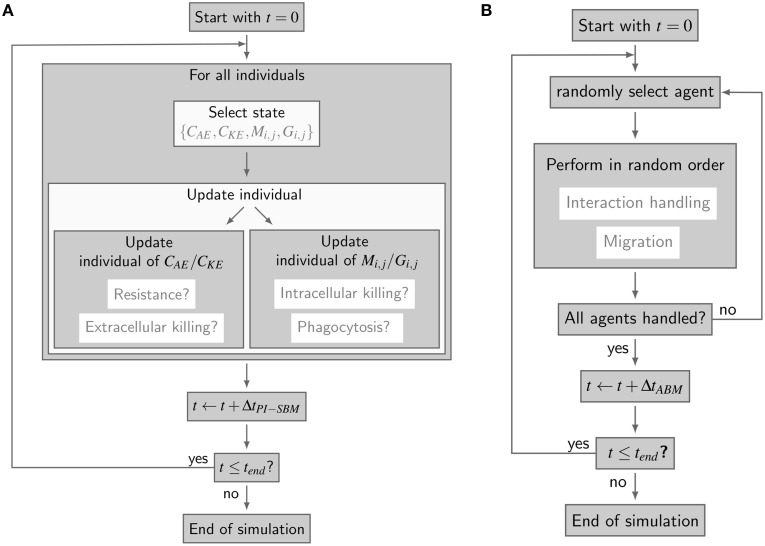
**Simulation algorithms of virtual infection models for whole-blood assays. (A)** Flow-chart of the non-spatial PI-SBM simulation algorithm. In each time-step Δ*t*_*PI*-*SBM*_, all individuals are tested for possible state transitions. Individuals of extracellular alive and killed *C. albicans* states, i.e., *C_AE_* and *C_KE_*, respectively, are tested for becoming resistant and for extracellular killing. Individuals of immune cell states (*M*_*i*, *j*_ or *G*_*i*, *j*_) are tested for phagocytosis of *C. albicans* and for intracellular killing. **(B)** Flow chart of the spatial ABM simulation algorithm. In each time-step Δ*t_ABM_*, the migration and interaction handling is performed in random order for every randomly chosen agent.

The simulation algorithm for the time-evolution of the PI-SBM is implemented in C++ that is available upon request. In Figure 2A, the simulation algorithm is schematically depicted and can be compared to the simulation algorithm of the P-SBM in Supplementary Figure 1. We simulate a blood sample of 1 ml containing 5 × 10^5^ monocytes, 5 × 10^6^ PMN and 1 × 10^6^ *C. albicans* cells that are initially extracellular and alive. In each time-step, which we set to Δ*t*_*PI*−*SBM*_ = 1 min, the algorithm tests for each individual cell in the system whether or not it does undergo a state transition. To this end, a cell is first randomly selected by sampling its relative frequency of occurrence among all cell types in the system. Next, the state of this cell is updated using a random selection procedure for the one transition in this time-step that the cell can possibly make among all currently enabled transitions. Once the type of transition between states *s* and *s*′ with rate *r_s→s′_* is selected, it will be executed with probability *P*_*s*→*s*′_ = *r_s→s′_* Δ*t*_*PI*−*SBM*_ and the system is updated accordingly. Table 1 provides an overview of the transition rates for all possible state transitions of the model. After testing all individuals in the system for performing a state transition, the simulation time is advanced by one time-step and the whole procedure is repeated until the total simulation time is reached. Note that, since the ratio of the number of immune cells over the number of pathogenic cells is larger than five, the simulation run time of the PI-SBM is significantly increased compared with the P-SBM.

**Table 1:** **Rates of state transitions in the non-spatial PI-SBM**.

**Transition rate**	**Description**	**State transition**
ϕ*_M_*	Phagocytosis by monocytes	*M*_*i*, *j*_ + *C_AE_* →*M*_*i*+1,*j*_ *M*_*i*, *j*_ + *C*_*KE*_ →*M*_*i*, *j*+1_
κ*_M_*	Intracellular killing by monocytes	*M*_*i*, *j*_ → *M*_*i*−1,*j*+1_
ϕ*_G_*	Phagocytosis by PMN for first-time phagocytosis event	*G*_0,0_ + *C*_*AE*_ →*G*_1,0_
		*G*_0,0_ + *C*_*KE*_ →*G*_0,1_
ϕ*G*⋆	Phagocytosis by PMN for repeated phagocytosis events	*G*_*i*, *j*_ + *C*_*AE*_ →*G*_*i*+1,*j*_
		*G*_*i*, *j*_ + *C*_*KE*_ →*G*_*i*, *j*+1_
κ_*G*_	Intracellular killing by PMN	*G*_*i*, *j*_ →*G*_*i*−1,*j*+1_
κ_*EK*_(*t*)	Extracellular killing by antimicrobial peptides released by first-time PMN phagocytosis with decreasing activity	*C*_*AE*_ →*C*_*KE*_
	Rate depends on the activity of antimicrobial peptides (κ_*EK*_) and the decay of their antimicrobial activity (γ) as defined in Hünniger et al. (2014)	
ρ	Resistance against phagocytosis and/or killing	*C*_*AE*_ →*C*_*AR*_,
		*C*_*KE*_ →*C*_*KR*_

*For details see (Hünniger et al., 2014)*.

### 2.2. Spatial agent-based model

**Figure 3:**
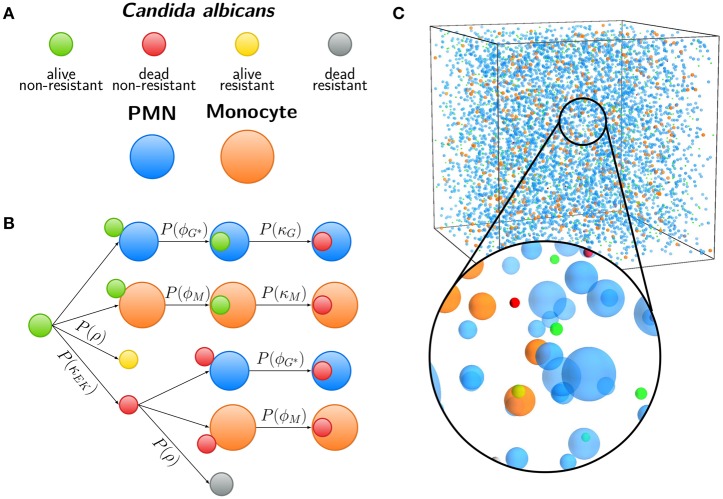
**Agent-based model (ABM) of the human whole-blood infection assay. (A)** Agents used in the ABM are *Candida albicans* cells in the four states (i) alive and non-resistant (green), (ii) dead and non-resistant (red), (iii) alive and resistant (yellow), and (iv) dead and resistant (gray). Furthermore, granulocytes (polymorphonuclear neutrophils, PMN) and monocytes are shown in blue and orange, respectively. **(B)** Schematic overview of examples for interactions in the ABM. Each arrow indicates the execution of an interaction event, either depending on spatial contact between different cells or by a contact-independent process. *C. albicans* cells that are in extracellular space and alive (*C_AE_*) or killed (*C_KE_*) can become resistant with probability *P*(ρ). After spatial contact, alive or killed *C. albicans* cells can be phagocytosed by PMN or monocytes with probabilities *P*(ϕ*_G_*|ϕ_G^*^_) or *P*(ϕ*_M_*), respectively. Intracellular alive *C. albicans* cells are killed with probabilities *P*(κ*_G_*) or *P*(κ*_M_*) depending on the type of phagocyte. **(C)** Visualization of the three-dimensional cuboid environment of the ABM that corresponds to 1 μl of the whole-blood infection assay, containing 5000 PMN, 500 monocytes, and 1000 *C. albicans* cells. The time-evolution of the simulated infection scenario can be viewed in Supplementary Video 1.

The spatial virtual infection model for *C. albicans* in human blood is realized using an ABM approach. This model is implemented in C++ based on a previously established framework of Pollmächer and Figge (2014) and is the spatial counterpart of the non-spatial PI-SBM introduced in Section 2.1. The C++ source code of the ABM simulation algorithm is available upon request. In the ABM, the two types of immune cells—monocytes and PMN—as well as the pathogenic *C. albicans* cells are incorporated as virtual objects. These virtual objects are agents that are characterized by a spherical morphology with the physiological diameters of *d_M_* = 16 μm for monocytes, *d*_*G*_ = 13.5 μm for PMN (Mak and Saunders, 2011) and *d_C_* = 7 μm for *C. albicans* (Mendling, 2006) (see Figure 3A) and that can migrate and interact with each other on encounter in the three-dimensional spatial environment (see Figure 3B). We impose a cuboid environment with an edge length of 1000 μm representing 1 μl blood and use *random periodic* boundary conditions for the cuboid, i.e., an agent which leaves the environment at some boundary point is deleted from the system and a new agent with identical properties re-enters the environment at some other randomly chosen boundary point. The cuboid environment is represented as a continuous space, i.e., allowing agents to move in a manner that is more realistic than could be captured by a lattice-based approach. This advantage is accompanied by the drawback that well-defined neighborhood relations as naturally existing between neighboring sites on a lattice are not present in continuous space representations. However, in order to efficiently determine cell–cell encounters, we use a neighborhood list method, which reduces the computational complexity to a close-to linear dependency on the number of agents in the system (Rapaport, 1996). At time point *t* = 0, agents are initialized with all *C. albicans* cells being in the state alive-and-extracellular. The time-evolution of the system is simulated by the random selection method (Skvoretz, 2002; Figge, 2005) that handles the migration and interaction of agents per time-step Δ*t* in a random fashion (see Figure 2B).

We use ratios in cell numbers that are equivalent to those in the PI-SBM, where 1 μl of blood contains 5 × 10^3^ PMN, 5 × 10^2^ monocytes and 1 × 10^3^ *C. albicans* cells, i.e., in total 6.5 × 10^3^ cells. Viewing cells as interacting point particles, an average volume of $v\approx \frac{1}{6.5}\times 10^{6}\mu {\text{m}}^{\text{3}}$ can be attributed to each cell, implying an average distance of $l\approx v^{1/3}\approx 55\mu \text{m}$ between immune cells and *C. albicans* cells. Even though this distance is clearly larger than the diameters of these cells, *l*≫ *d_M_*, *d_G_*, *d_C_*, we assume that the migration behavior of immune cells and *C. albicans* cells in blood resembles a random walk of agents without directional persistence. This assumption is based on the fact that the total number of erythrocytes in human blood ranges from 4× 10^6^ − 6× 10^6^ cells/μl (McClatchey, 2003). Estimating the total number of cells in 1 μl of blood to be about six millions, an average volume of $v_{c}\approx \frac{1}{6}\times 10^{3}\mu {\text{m}}^{\text{3}}$ can be attributed to each cell, implying a mean free path of $l_{fp}\approx v_{c}^{1/3}\approx 5\mu \text{m}$ between point particles. This distance is not only clearly smaller than the distance between immune cells and *C. albicans* cells, *l_fp_* ≪ *l*, but also smaller than the diameters of erythrocytes, *C. albicans* cells as well as of the immune cells under consideration. It can be concluded that cells are not migrating with directional persistence in blood, because frequent collisions with the overwhelming number of erythrocytes will induce diffusive migration of cells with diffusion coefficients in whole-blood that can be very different for the different cell types. This is a consequence of the fact that monocytes and PMN perform active migration, whereas *C. albicans* cells are immotile due to the complete lack of cellular organelles for motility (Margulies and Schwartz, 1998) and its movement in whole blood is only passive.

Even though blood is a non-Newtonian fluid, i.e., showing pseudoplastic properties with variable viscosity depending on the exerted shear stress in capillaries of different sizes (Fahraus and Lindqvist, 1931), the experimental setup of the whole-blood infection assay is such that the viscosity as well as the temperature in the mildly stirred test tube remain constant (Hünniger et al., 2014). Therefore, the Stokes-Einstein equation (Einstein, 1905) can be applied to infer the diffusion coefficient *D_C_* for the passive movement of *C. albicans* cells. Based on a whole-blood viscosity of about η ≈ 4 *mPas* (Rosenson et al., 1996), Boltzmann constant *k_B_* and temperature *T* = 37 °C (Hünniger et al., 2014), this yields the relatively small diffusion coefficient *D_C_* = *k_B_T*/(3πη *d_C_*) ≈ 1 μm^2^/min. In contrast, the active migration of monocytes and PMN requires to estimate their diffusion coefficients numerically.

The time-step Δ*t_ABM_* for simulations in the ABM has to be chosen such that a smooth migration of cells is sampled in time. In order to ensure this, we require that during one time-step Δ*t_ABM_* cells do not migrate further than a certain distance, which we set to equal the mean free path *l_fp_* = 5 μm:

(6) $$\Delta t_{ABM}=\frac{l_{fp}^{2}}{6D_{max}}.$$

Here, *D_max_* ≡ max{*D_C_*, *D_M_*, *D_G_*} denotes the largest out of the three diffusion coefficients for *C. albicans* cells (*D_C_*), monocytes (*D_M_*), and PMN (*D_G_*). Since it can be expected that the active migration of immune cells is associated with diffusion coefficients *D_M_* and *D_G_* with *D_M_*, *D_G_*≫ 1 μm^2^/min in the whole-blood infection assay, it follows from Equation (6) that the time-step in the ABM will be much smaller than in the state-based model PI-SBM: Δ*t_ABM_* ≪ Δ*t*_*PI*−*SBM*_ = 1 min. Moreover, stochasticity in the ABM requires that each simulation has to be repeated multiple times, resulting into relatively high computational costs compared with the PI-SBM, in particular, if we would have envisaged to estimate each model parameter instead of following the strategy of a bottom-up modeling approach.

Computational costs associated with parameter estimation in the ABM can be significantly reduced by making use of the previously estimated rates of state transitions in the state-based model PI-SBM (see Section 2.1 and Table 1). In the course of a simulation, migrating cells in the ABM may either spontaneously undergo state transitions or interact with each other upon spatial contact. In Figure 3C, we present a schematic overview of processes that occur according to defined rules associated with certain probabilities. It is important to note that, due to the spatial aspects that are captured by the ABM but not the PI-SBM, we have to distinguish between processes that are *contact-dependent* and *contact-independent*.

For *contact-independent* processes—such as intracellular and extracellular killing as well as the occurrence of *C. albicans* resistance against phagocytosis and/or killing—the conversion of rates from the PI-SBM to the ABM is straightforward. Since these processes are not determined by any spatial requirements, a simple re-scaling is performed. For example, *C. albicans* cells become resistant in the PI-SBM with probability *P_PI−SBM_*(ρ) = ρ Δ*t*_*PI−SBM*_. In the ABM, where the resolution of time is set by the time-step Δ*t_ABM_* ≪ Δ*t*_*PI−SBM*_, we check in each time-step with probability

(7) $$P_{ABM}(\rho )=P_{PI-SBM}(\rho )\frac{\Delta t_{ABM}}{\Delta t_{PI-SBM}}$$

whether this process occurs.

In contrast, *contact-dependent* processes in the ABM are characterized by the requirement that two cells have to get into spatial contact first, before such a process—for example, a phagocytosis event of a *C. albicans* cell by a monocyte with transition rate ϕ*_M_*—can take place. In the PI-SBM, spatial contact is not explicitly modeled; rather, the interaction partner for each monocyte is randomly chosen once per time-step Δ*t*_*PI*−*SBM*_. The associated probability is determined by the time-dependent ratio of non-resistant fungal cells over the sum of extracellular fungal cells and immune cells. Once an interaction partner was chosen, the phagocytosis event itself occurs with probability *P_PI−SBM_*(ϕ*_M_*) = ϕ*_M_* Δ*t*_*PI*−*SBM*_ in the PI-SBM. Correspondingly, in the ABM, we request that this process takes place with the same probability,

(8) $$P_{ABM}(\phi_{M})=P_{PI-SBM}(\phi_{M}),$$

on every encounter between a monocyte and a *C. albicans* cell. This correspondence of event probabilities for the two modeling approaches imposes a condition on the spatial dynamics of cells, i.e., on the values of the diffusion coefficients in the ABM and by that on the time-step Δ*t_ABM_* (see Equation 6). For optimal migration parameters, i.e., parameters that result in good agreement with the experimental data, it is expected that measurement of the associated phagocytosis rate in the ABM coincides with the corresponding rate from the PI-SBM.

### 2.3. Parameter estimation

#### 2.3.1. Simulated annealing

**Figure 4:**
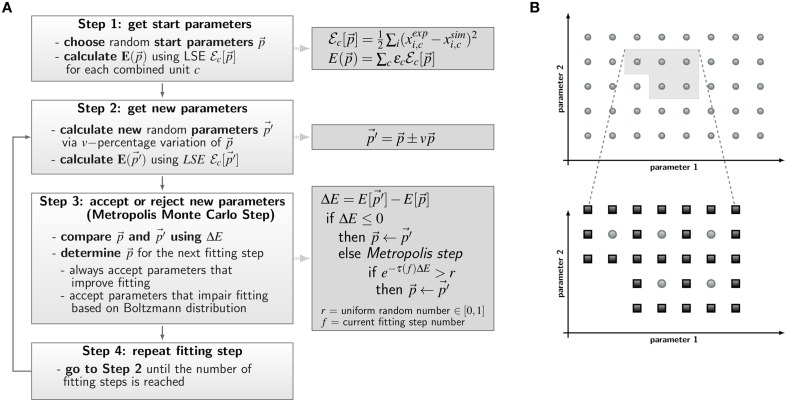
**Concepts of parameter estimation algorithms for non-spatial state-based models (SBM) and spatial agent-based models (ABM). (A)** Flow chart of parameter estimation by the global optimization method Simulated Annealing based on the Metropolis Monte Carlo (SA-MMC). Light gray boxes describe Steps 1–4 of the algorithm; equations and pseudo code applied in these steps are provided in the dark gray boxes. **(B)** Schematic overview of the local optimization method *adaptive grid search algorithm* used for estimation of ABM migration parameters. Simulations are performed for sets of parameters on a regular grid in a predefined area of the search space and are evaluated by the *least squares error* (LSE). The initial grid (upper panel) contains sets of parameters with smallest LSE (light gray area) and this area is refined for a more accurate identification of the optimal parameter set (lower panel). Here, light gray dots represent parameter configurations from the first refinement level and dark gray squares represent parameter configurations of the subsequent refinement level.

The *a priori* unknown transition rates of the PI-SBM are determined by the method of Simulated Annealing based on the Metropolis Monte Carlo scheme (SA-MMC) that is depicted in Figure 4A. This optimization method randomly explores the parameter space of transition rates to find the global minimum of the fitting error, i.e., the most suitable parameter set that produces the best fit of the simulation to the experimental data obtained from the whole-blood infection assay.

The parameter estimation algorithm starts with a randomly chosen set of parameter values within the interval of [0,1] per minute, represented by the vector $\vec{p}$, and calculates the resulting time-evolution of state occupations from the simulation algorithm of the PI-SBM (see Section 2.1). To score the simulation result for a particular set of parameters, we combined different kinetics of the PI-SBM, referred to as *combined units*, which are identical with the experimental kinetics measured in the whole-blood infection assay (see Section 2.1). In this way, the experimental kinetics can be directly compared with the combined units *c* obtained from the model simulation, which is then scored by calculating the least-squares error (LSE) at experimental data points *k* as the weighted sum over *c*:

(9) $$E[\vec{p}]=\sum_{c}\epsilon_{c}\frac{1}{2}\sum_{k}{\left(x_{k,c}^{dat}-x_{k,c}^{sim}[\vec{p}]\right)}^{2}.$$

Here, ϵ_*c*_ is adjusted as to fit each combined unit comparably well to the experimental data. The same values for ϵ_*c*_ were used in the PI-SBM and the ABM and are given in Supplementary Table 1. Next, the parameter set $\vec{p}$ is randomly varied within a pre-defined neighborhood of 10% variation, leading to a new set of parameter values, ${\vec{p}}^{\;\prime }$, as indicated in Figure 4A, Step 2. Subsequently, the simulation of the PI-SBM is performed again for parameter values ${\vec{p}}^{\;\prime }$ and the corresponding score *E*[${\vec{p}}^{\;\prime }$] is calculated. Whether the new simulated data will be accepted or rejected is decided by applying the MMC scheme that is depicted in Figure 4A, Step 3. The probability to accept worse parameter values is influenced by τ(*f*), representing the “inverse system temperature” in a SA process. The simulation of the annealing process involves a gradual decrease of the system temperature with progressed fitting, i.e., τ(*f*) is increased with the number of performed fitting steps *f* (see Supplementary Information 2.1).

After performing a total number of fitting steps, the fitting algorithm is repeated starting from a newly chosen random parameter set. This is done for a certain number of runs and the set of parameters with the minimal fitting error ($\vec{p}$_min_) is saved from each fitting process. The mean values of the parameter values and their standard deviations are computed over all runs to determine the robustness of the estimated parameters.

We repeatedly perform the parameter estimation procedure for different system sizes in terms of the total number of individual cells. In doing so, the system size is stepwise increased by factors of ten, which is associated with increasing computing time for the model simulation but is partly compensated by a decrease in the number of fitting steps to avoid computational overload (see Supplementary Table 2). We start the estimation algorithm with a low number of individuals and a large number of fitting steps. The resulting parameter values are subsequently used as start parameter values for the system with next-higher number of individuals, i.e., for a 10-fold larger system. This procedure is repeated until a system size is reached where the number of individuals correspond to the measured numbers of PMN (about 5 × 10^6^) and monocytes (about 5 × 10^5^).

#### 2.3.2. Adaptive regular grid search

As described in Section 2.2, probabilities for state transitions in the ABM of the whole-blood infection assay can be derived from the interaction rates of the PI-SBM. This reduces the space of parameters that has to be searched in the process of parameter estimation, leaving only two migration parameters—i.e., the diffusion coefficients *D_M_* and *D_G_*, respectively, for monocytes and PMN—to be calibrated. However, even for a reduced parameter search space, there still is need for a calibration strategy that keeps the number of ABM simulations within limits, because simulating stochastic processes requires sufficient numbers of repetitions in order to obtain numerical results that are statistically sound.

We apply the *adaptive regular grid search algorithm* (Powell, 1998) to search iteratively for a local optimum in the parameter space (see Figure 4B). Motivated by biological constraints this is done for a pre-defined region of the parameter space. This region is represented on a regular grid and for each grid point the ABM is simulated with the corresponding set of parameter values. Afterwards, simulations are evaluated with the least-squares error (LSE), scoring deviations between the simulation results and the experimental data for all combined units *c* = {*C_K_, C_A_, C_E_, C_M_, C_G_*} (see Section 2.1 and Equation 9). The values for the LSE are used to determine the adaptive refinement of the grid before the next iteration step, where intermediate grid points are calculated by bisection of the grid constant for the sets of parameters with lowest LSE. This imposes a grid refinement that ensures a more detailed scanning of the parameter space in relevant regions and defines the refinement level. The initial grid constant and the number of refinement steps determine how fine-grained the parameter space is represented by grid points and their values have to be chosen depending on the LSE landscape.

We further decrease computational costs associated with parameter estimation in the ABM by system scaling. Thus, similar to the procedure applied for the state-based model PI-SBM, we first scan the parameter space with a small system of 1/5μl blood and subsequently re-scan the relevant parameter region with the system of 1 μl blood as defined in Section 2.2.

## 3. Results

### 3.1. Quantification of the immune response by the state-based model

We quantified innate immune mechanisms in human whole-blood assays of infection with the pathogenic fungus *C. albicans* using a SBM. To this end, we modified a previously introduced SBM, referred to as P-SBM. This model was derived with the focus on state transitions of the pathogen (P) that may be induced by immune cells. However, immune cells in the P-SBM were only effectively modeled and not explicitly account for as individual cells (Hünniger et al., 2014). In the present work, we modified the P-SBM to model the interaction with individual immune cells—monocytes and granulocytes (PMN)—in detail. Since the focus of this model is on state transitions of both pathogen (P) and immune cells (I), we term this model PI-SBM. For reasons of comparison with the P-SBM, we used the same experimental data as in Hünniger et al. (2014) to quantify innate immune mechanisms by estimating the transition rates that yield the best fit to the data. Specific combinations of *C. albicans* states, referred to as *combined units*, were introduced that are directly related to different *C. albicans* populations measured over 4 h post-infection in experiment. As explained in detail in the Materials and Methods Section, the combined units include all extracellular *C. albicans* cells (*C_E_*), *C. albicans* cells that are phagocytosed, respectively, by monocytes (*C_M_*) or by granulocytes (*C_G_*). Furthermore, all killed and alive *C. albicans* cells are given by the combined units *C_K_* and *C_A_*, respectively. The manually adjusted scores ϵ_*c*_ of combined units *c* are given in Supplementary Table 1. We simulate a blood sample of 1 ml containing 5 × 10^5^ monocytes, 5 × 10^6^ PMN and 1 × 10^6^ *C. albicans* cells that are initially extracellular and alive.

**Figure 5:**
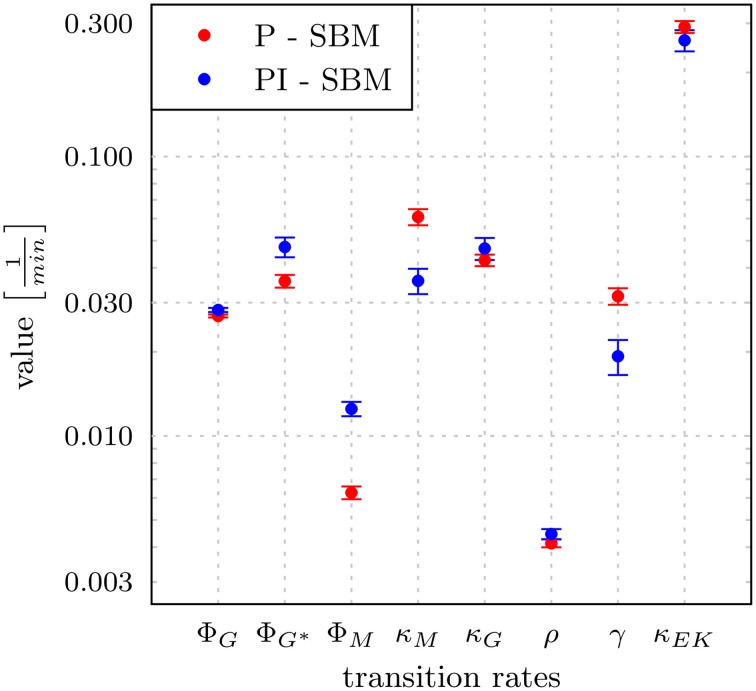
**Transition rates obtained from the model calibration to experimental data of the whole-blood infection assay**. The results for the modified state-based model PI-SBM are compared to the P-SBM (Hünniger et al., 2014). The values are compared for the rate of phagocytosis by monocytes (ϕ*_M_*), and by PMN on initial and subsequent events (ϕ_*G*_, ϕ_G^*^_), rate of killing by monocytes (κ*_M_*) and PMN (κ*_G_*), rate of acquiring resistance against phagocytsis and/or killing (ρ) as well as the values of parameters for extracellular killing (γ, κ*_EK_*). Error bars correspond to standard deviations.

To estimate the values of transition rates in the PI-SBM that yield the best fit to experimental data, i.e., the fit with the smallest least squares error (LSE), we applied the method of SA-MMC scheme (for details see Section 2.3.1). In Figure 5, the resulting transition rates of the PI-SBM are compared with those previously obtained within the P-SBM (for a quantitative comparison see also Supplementary Tables 3, 4). The direct comparison between the P-SBM and PI-SBM revealed that most transition rates are quantitatively similar in the two models.

The largest deviations in the values of transition rates between the two models were observed for the phagocytosis rate of monocytes (ϕ*_M_*) and the killing rate of monocytes (κ*_M_*). This was further investigated by performing the parameter estimation for the PI-SBM again, where only ϕ*_M_* and κ*_M_* were randomly varied while all other rates were kept fixed. We performed 50 runs and obtained very different standard deviations for these transition rates: while the standard deviation of ϕ*_M_* was only 4%, this was 16% in the case of κ*_M_*. We conclude that the PI-SBM is generally robust in all transition rates, except for κ*_M_* that is also not directly determined by the data, because alive and killed *C. albicans* cells in phagocytes were not distinguished in these experiments. Similar observations were made for the P-SBM, where it was shown that variations in κ*_M_* did not lead to significant differences in the fitting error (Hünniger et al., 2014).

**Figure 6:**
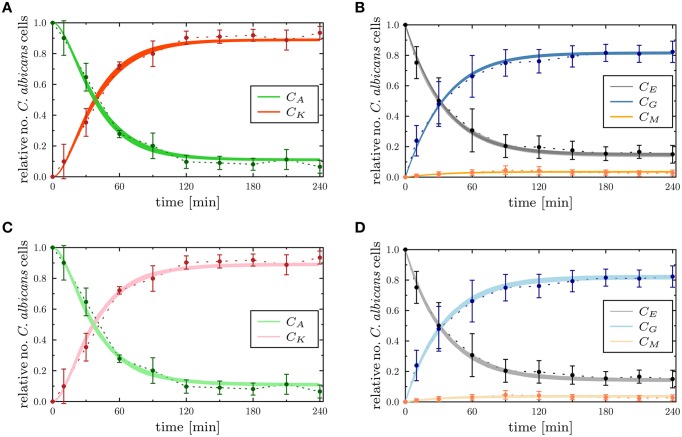
**Comparison of the time-evolution for the combined units from the experimental whole-blood infection assay (dotted lines as a guide for the eye) with the PI-SBM in (A,B), and the ABM in (C,D)**. In **(A,B)**, the thickness of the solid lines represents the standard deviation of the PI-SBM simulation results as obtained from 50 simulations for normally distributed transition rates as given in Supplementary Table 3. The thickness of the solid lines in **(C,D)** represents the standard deviation obtained by 30 simulations of the stochastic ABM. Time-evolution of killed (*C_K_*) and alive (*C_A_*) *C. albicans* cells are depicted in **(A,C)**, and the dynamics of *C. albicans* cells that are in extracellular space (*C_E_*), phagocytosed by monocytes (*C_M_*) and PMN (*C_G_*) are shown in **(B,D)**.

To determine the impact of variations in the transition rates on the kinetics of the combined units in the PI-SBM, we performed 50 simulations with transition rates that were randomly sampled within their respective standard deviations. The kinetics of individual sub-populations are plotted in Supplementary Figure 2 while the results for the combined units are given in Figure 6. It can be seen that the simulated combined units agree well with the corresponding experimental data. In particular, the resulting kinetics of the PI-SBM revealed that 4 h post-infection 82% *C. albicans* cells were phagocytosed by PMN, whereas only 4% *C. albicans* cells were phagocytosed by monocytes. Furthermore, PMN play a major role in the immune response, because these phagocytes are associated with 97% of all killed *C. albicans* cells (see Supplementary Figure 2A). This is achieved either directly, via phagocytosis and intracellular killing (66.5%) of the pathogen, or indirectly by the release of antimicrobial peptides on a pathogen's first event of phagocytosis (30.5%) (see Supplementary Figure 2H). Four hours post-infection, most *C. albicans* cells were killed (89%) while a minority of 11% cells were extracellular and became resistant against killing and phagocytosis. These results are in quantitative agreement with those obtained previously for the P-SBM (Hünniger et al., 2014).

### 3.2. Predictions on monocytopenia and neutropenia from PI-SBM

**Figure 7:**
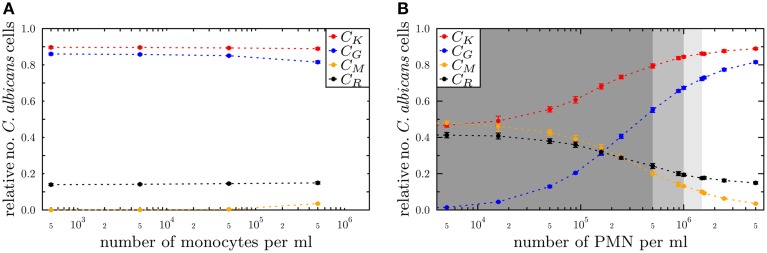
**Simulation results of the PI-SBM with different immune cell numbers at 4 h post-infection for the conditions (A) monocytopenia and (B) neutropenia**. The relative numbers of *C. albicans* cells of killed cells (*C_K_*), phagocytosed cells in monocytes (*C_M_*) and in PMN (*C_G_*) as well as cells that became resistant (*C_R_*) against killing and/or phagocytosis are depicted for different numbers of monocytes and PMN. The number of **(A)** monocytes and **(B)** PMN in the simulations are reduced separately, starting from physiological concentrations of 5×10^5^ /ml monocytes and 5×10^6^ /ml PMN down to vanishing concentrations. In **(B)**, the light gray region represents the range of light neutropenia (<1.5× 10^6^ PMN per ml), medium gray region represents the range of moderate neutropenia (<1× 10^6^ PMN per ml) and dark gray region represents the range of severe neutropenia (<5× 10^5^ PMN per ml).

The state-based model PI-SBM opens the possibility to study the dependence of the immune response against *C. albicans* on the number of PMN and monocytes in blood. Simulating the virtual infection scenario with the previously estimated parameters (see Supplementary Table 3), we considered various cases of immune cell deficiencies. The model predictions at 4 h post-infection and for gradual decreases in the immune cell numbers are presented in Figure 7 for the cases of monocytopenia and neutropenia separately.

We found, as expected from the above quantification of the immune response, that monocytopenia is not a critical condition with regard to *C. albicans* infections: deficiency of monocytes and even their complete absence was fully compensated by PMN-mediated killing. In fact, patients with monocytopenia have not been reported to develop systemic candidiasis to date (Lionakis, 2014). The situation is extremely different in the case of neutropenia. In the absence of PMN, the number of killed *C. albicans* cells is predicted to decrease from about 89% under physiological conditions down to 45%, i.e., *C_K_* = 89% for 5 × 10^6^ PMN and *C_K_* = 45% for ≤5×10^3^ PMN (see Figure 7B). Monocytes compensated PMN deficiency by phagocytosis of *C. albicans* cells only partly, where the fraction increased from 3% under physiological conditions up to 48%. However, 42% of the *C. albicans* cells acquired resistance against killing and/or phagocytosis, resulting from the combined effect of absent PMN phagocytosis and extracellular killing that is normally mediated by PMN release of antimicrobial peptides.

For a decrease in PMN number by one order of magnitude from physiological conditions, we found that monocytes can sustain the immune response fairly well. In this case, the fraction of killed *C. albicans* cells was still 79% and the phagocytosis by monocytes and PMN reached, respectively, 20% and 55% of *C. albicans* cells. A significant deterioration of the immune response was observed for PMN concentrations below 5 × 10^5^ cells/ml (see Figure 7). Interestingly, this concentration was reported to mark the transition from moderate to severe neutropenia (Munshi and Montgomery, 2000), which is a condition that is known to be associated with high risks for candidemia in cancer patients (Lunel et al., 1999; Alangaden, 2011).

### 3.3. Agent-based model captures immune response in time and space

State-based models (SBM) do not account for any spatial aspects. For example, cells in the PI-SBM do not actually migrate during the immune response and, therefore, do not have to get into contact before a phagocytosis event can take place. In contrast, agent-based models (ABM) do capture spatial aspects in a defined environment. Applying a bottom-up modeling approach, we implemented an ABM that is—apart from its spatial aspects—the exact analog of the non-spatial PI-SBM. As depicted in Figure 1, all transition rates that were previously estimated for the PI-SBM were fed into the ABM (see Section 2.2 for details). The only parameters left to estimate were those related to cell migration, which in the dense cell system of the whole-blood assay resembles a random walk. In particular, while the diffusion coefficient associated with the passive movement of *C. albicans* cells could be inferred from the Stokes-Einstein equation to be *D*_*C*_ ≈ 1 μm^2^/min, the active migration behavior of immune cells requires a rigorous parameter estimation of the diffusion coefficients *D_M_* and *D_G_* for monocytes and PMN, respectively.

It should be noted that, even in the case of low-dimensional parameter spaces, the estimation of parameters for ABM generally turn out to be computationally intensive. This is a consequence of the fact that ABM simulate the interactions between thousands of agents in continuous space as stochastic processes. To simultaneously facilitate an increase in model complexity and a decrease in computational expense for parameter estimation, we applied the local optimization algorithm *adaptive regular grid search*. This algorithm compares ABM simulations by evaluating the least squares error (LSE) regarding the experimental data of the whole-blood infection assay. Stochastic effects of the ABM were investigated by comparing simulation results for a fixed set of parameter values with varying number of *in silico* replicates. Using 100 *in silico* replicates as a reference for the mean value of the LSE, we generally observed for relevant parameter sets, i.e., parameter sets that yield reasonable agreement with the experimental data, that relative variations in the mean LSE were already well below 10% for 30 *in silico* replicates. Therefore, we set the number of *in silico* replicates to 30 throughout the whole parameter space.

**Figure 8:**
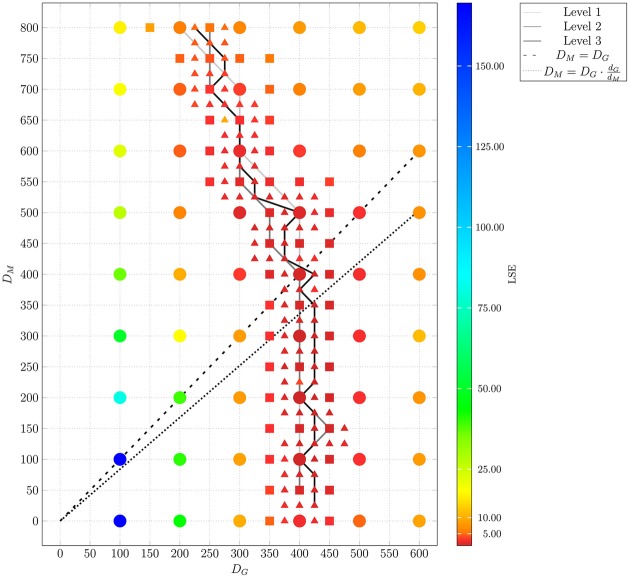
**Result of ABM parameter estimation by *adaptive regular grid search***. The diffusion coefficients for monocytes, *D_M_*, and PMN, *D_G_*, were scanned within the regime that was determined by parameter scanning for the small ABM (1/5 μl blood). At each grid point, 30 ABM simulations were performed for the large system (1 μl blood) and the mean least squares error (LSE) is depicted. By determining the path of minimal LSE values and adding grid points around this path by adaptive bisection, three refinement levels are considered. Dots represent grid points of the first refinement level, squares represent grid points of the second refinement level, and triangles represent grid points of the third refinement level. The paths of minimal LSE values for the first, second and third refinement level are traced by light gray, medium gray, and dark gray lines, respectively.

The *adaptive regular grid search* algorithm searches the space of *D_M_* and *D_G_* on a pre-defined grid of diffusion coefficients, 0 < *D_M_*, *D_G_* < 800 μm^2^/min. This range for the diffusion coefficients implies that the time step Δ*t_ABM_* varies between 5.2 × 10^−3^ min ≤ Δ*t_ABM_* ≤ 4.2 min (see Equation 2.3.2). As described in Section 2.3.2, we started with a relatively coarse grid of step size 100 μm^2^/min and computed at each grid point the LSE by comparing the experimental data with a small ABM system, i.e., representing 1/5 μl of blood (see Supplementary Figure 3). These results were used to determine the regime of parameters in which the parameter estimation was continued for the large ABM system simulating 1 μl of blood. The parameter regime was determined by the rectangle that contains all pairs of diffusion coefficients (*D_G_*, *D_M_*) for which the LSE values were found to be minimal from separately varying each parameter. The corner points of this rectangle were (*D_G_*, *D_M_*) = (100,0) μm^2^/min and (*D_G_*, *D_M_*) = (600,800) μm^2^/min (see gray region in Supplementary Figure 3). Subsequently, the grid was refined based on simulations of the large ABM by determining the path of minimal LSE values and adding grid points around this path by adaptive bisection. After simulation of the ABM for parameter sets corresponding to the added grid points, the procedure of grid refinement was repeated. This can be seen in Figure 8, where we plot a map of the LSE landscape together with the paths of minimal LSE values for each level of refinement. It was observed that the course of these paths covers a relatively broad range of diffusion coefficients for monocytes, *D_M_*, whereas this is a fairly narrow range of *D_G_*-values for PMN.

**Figure 9:**
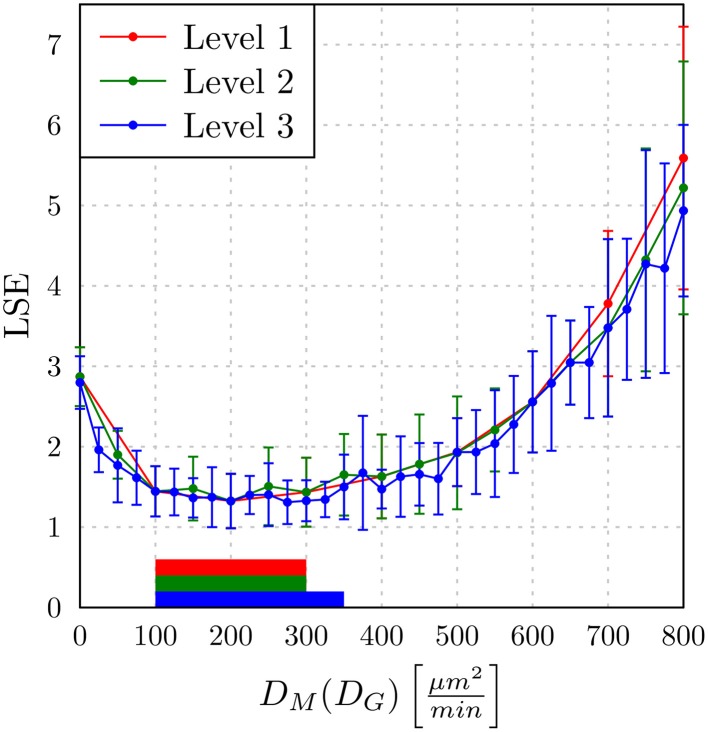
**The least squares error (LSE) of the paths of grid points along the diffusion coefficients for monocytes (*D_M_*) as a function of the minimal diffusion coefficient for PMN (*D_G_*): *D_M_*(D_G_)**. Mean values and standard deviations were obtained from averaging over 30 ABM simulations. The paths of the first, second, and third refinement level are shown, respectively, as red, green, and blue lines (guide for the eyes). The horizontal bars indicate regions of diffusion coefficients with values comparable to the absolute LSE minimum of each refinement level. All values outside these regions are significantly different from the absolute LSE minimum (Wilcoxon rank sum test with *p* < 0.05).

In Figure 9, we present the LSE values as a function of *D_M_*(*D_G_*) along the paths of minimal LSE values for the three levels of refinement. From the third level of refinement we inferred the point of absolute LSE minimum to be located at (*D^min^*_*G*_,*D*^*min*^_*M*_) = (425,275) μm^2^/min. However, since the landscape of *D_M_*(*D_G_*) resembled an extended valley across neighboring data points, we performed a statistical analysis by applying the Wilcoxon rank sum test between the absolute LSE minimum and its neighboring points to check for significant differences between them. Imposing a *p*-value of *p* < 0.05 for significant difference, we obtained points with similar values of the LSE ranging in the interval *D_M_* = 100 μm^2^/min to *D_M_* = 350 μm^2^/min for monocytes and *D*_*G*_ = 400 μm^2^/min to *D*_*G*_ = 425 μm^2^/min for PMN (see Figure 8). These findings imply that the immune response in the whole-blood infection assay was much more sensitive to variations in the diffusion coefficients of PMN than of monocytes, emphasizing the dominant role of PMN over monocytes from the viewpoint of cell migration.

Our results are consistent with the absolute LSE minima of refinement level one and two, which were both at (*D^min^_G_*, *D^min^_M_*) = (400,200) μm^2^/min and that also belong to this interval (see Figure 9). Interestingly, while we expected that monocytes are less migratory active than PMN, i.e., restricting the relevant parameter regime in Figure 8 to the region below the dashed line, we also found that the interval around the absolute LSE minimum contains the parameter set (*D_G_*, *D_M_*) = (425,350) μm^2^/min. The ratio of these diffusion coefficients, *D_M_*/*D_G_* ≈ 0.82, resembles the value expected from the Stokes-Einstein equation (Einstein, 1905) implying *D_M_*/*D_G_* = *d_G_/d_M_* (dotted line in Figure 8). Taken together, we consider the diffusion coefficients (*D^min^*_*G*_, *D*^*min*^_*M*_) = (425,275) μm^2^/min to represent the immune cell dynamics reasonably well and use these values in our further analyses below.

Next, we compared the ABM simulation results for the absolute LSE minimum with those of the PI-SBM. These are plotted together with the experimental data of the whole-blood infection assay in Figure 6 and in Supplementary Figure 4 for the time evolution of *C. albicans* sub-populations. Thus, we found that both modeling approaches, the non-spatial SBM and the spatial ABM, yielded excellent agreement with the experimental data. Furthermore, we found that our simulation results obtained from the stochastic ABM were robust, which can be seen from the line thicknesses in Figures 6C,D representing the standard deviations obtained from 30 ABM simulations.

### 3.4. Predictions on hyper- and hypo-inflammation from ABM

**Figure 10:**
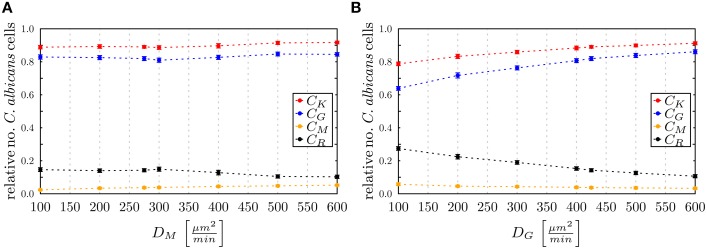
**Simulation results of the ABM at 4 h post-infection for varied diffusion coefficients around the absolute least squares error (LSE) minimum with (*D^min^*_*G*_,*D*^*min*^_*M*_) = (425,275) μm^2^/min for (A) monocytes keeping *D_G_* fixed and (B) PMN keeping *D_M_* fixed**. The relative numbers of *C. ablicans* cells of killed cells (*C_K_*), phagocytosed cells in monocytes (*C_M_*) and in PMN (*C_G_*) as well as cells that became resistant (*C_R_*) against killing and/or phagocytosis are depicted.

To investigate the impact of hyper- and hypo-inflammation associated with the dynamics of immune cells, we varied the diffusion coefficients of monocytes and PMN separately around the absolute LSE minimum (*D^min^*_*G*, *D*_*^min^_M_*) = (425,275) μm^2^/min. Keeping the diffusion coefficient *D_G_* fixed and varying the *D_M_* for monocytes between 100 μm^2^/min and 600 μm^2^/min, we observed at 4 h post-infection no substantial changes in the populations of killed, resistant and phagocytosed *C. albicans* cells (see Figure 10A). At extreme values *D_M_* > *D_G_*, a slight increase (decrease) in the number of killed (resistant) *C. albicans* cells was observed accompanied by a slight increase in the phagocytosis by both monocytes and PMN. This may be attributed to a stronger mixing of the cell system for high diffusion coefficients *D_M_*. In general, however, the immune response does not appear to be sensitive to this parameter, which is in agreement with the finding for monocytopenia that did not have a substantial impact on infection clearance (see Figure 7A).

In the opposite case, where *D_M_* was fixed and *D_G_* was varied between 100 μm^2^/min and 600 μm^2^/min, it was again observed that for increased values *D*_*G*_ > 425 μm^2^/min the impact on the immune response against *C. albicans* is only weak. In contrast, for decreased values *D_G_* < 400 μm^2^/min the immune response was strongly affected by the reduced migratory activity of PMN. This could be observed by a substantial increase (decrease) in the number of resistant (killed) *C. albicans* cells (see Figure 10B). In particular, for *D_G_* = 100 μm^2^/min the phagocytosis of *C. albicans* cells by PMN was reduced by more than 20% and the relative number of resistant *C. albicans* cells reached the value of 28%. Comparing this scenario with the condition of PMN deficiency (see Figure 7B), we found that this PMN paralysis resembles moderate to severe neutropenia associated with a relative number of about 20% and 30% of resistant *C. albicans* cells, respectively.

## 4. Discussion

In this study, we applied a bottom-up modeling approach to simulate an experimental infection assay for *C. albicans* in human blood. As illustrated in Figure 1, this approach combines different mathematical models with increasing complexity that build on one another. We started from a previously developed state based model (SBM), here referred to as P-SBM (Hünniger et al., 2014), that neglects all spatial aspects and describes the time-evolution of pathogens in different states—e.g., alive, phagocytosed and killed—during the early response of the innate immune system. To include the immune response mediated by monocytes and granulocytes (PMN), in this work we modified the P-SBM into a SBM that does as well-explicitly account for the immune cell states and is therefore referred to as PI-SBM. The rates of state transitions in the PI-SBM were estimated by comparison with experimental data (Hünniger et al., 2014) using the global optimization method *simulated annealing* based on the Metropolis Monte Carlo scheme (SA-MMC).

The resulting model kinetics of both SBM were found to be in quantitative agreement with experimental data and confirmed that PMN play the major role in the immune defense against *C. albicans* in human blood. This is indicative for the general validity of both models, despite the structural difference of the simulation algorithms regarding the level of detail at which immune cells are modeled. Furthermore, the PI-SBM allows making predictions on infection scenarios in patients with immune cell deficiencies, i.e., neutropenia and monocytopenia. Performing *in silico* experiments with varying numbers of either monocytes or PMN, revealed that loss of monocytes was mainly compensated by PMN. In contrast, decreasing PMN number lead to a strongly reduced immune response against *C. albicans* for PMN numbers below 5×10^5^ /ml (see Figure 7). Our quantitative prediction is substantiated by published data that account this PMN concentration as severe neutropenia (Munshi and Montgomery, 2000). It is also reported that neutropenia impairs the outcome of candidemia and is a risk factor, in particular, for cancer patients developing candidemia (Guiot et al., 1994; Bow et al., 1995; Lunel et al., 1999). From the quantitative agreement between predictions of the PI-SBM and reported findings for *C. albicans* infection, we attribute a high predictive potential to this virtual infection model that may be exploited in future studies, e.g., focusing on conditions of immune dysregulation and/or comparing the impact of different pathogens. The possibility to quantify functional alteration of immune cells rather than pure numerical aberrations is of particular interest in this regard.

In order to account for spatial aspects of the immune response, we extended the SBM to an agent-based model (ABM), where cells are simulated as agents that can migrate in continuous three-dimensional space and can interact with each other on encounter in space. Applying the bottom-up modeling approach, we made use of the rates that were determined by fitting the PI-SBM to the experimental data and estimated the diffusion coefficients of immune cells in blood (see Figure 1). Due to high computational costs of ABM simulations, applying the global optimization method SA-MMC was no realistic option and we chose the computationally affordable local optimization method *adaptive regular grid search*. This method searches for the optimum within a pre-defined parameter space, which in the present case was a two-dimensional space spanned by the diffusion coefficients for monocytes and PMN. In contrast, applying SA-MMC was beneficial in the case of PI-SBM for three reasons: (i) the parameter space was eight-dimensional, (ii) limitations of the parameter space would have been difficult to motivate biologically, and (iii) computational costs for repeated simulations were still acceptable due to the neglect of spatial aspects.

As live cell imaging in whole-blood assays cannot yet be performed today, computer simulations are the only tool to predict diffusion coefficients of immune cells. Parameter estimation of the ABM predicted intervals for the diffusion coefficients that yielded quantitatively comparable results. For monocytes this interval, *D_M_* = 100 μm^2^/min to *D_M_* = 350 μm^2^/min, was substantially broader than for PMN with *D_G_* = 400 μm^2^/min to *D*_*G*_ = 425 μm^2^/min, indicating the importance of fine-tuned PMN motility.

Furthermore, by varying the diffusion coefficients of the immune cells, we demonstrated the impact of hyper- and hypo-inflammation in immune dysregulation. In general, reducing (increasing) immune cell motilities around optimal values reduced (increased) the number of interaction events between cells and by that the phagocytosis of *C. albicans* cells. In the case of PMN, reduction of cell motility and phagocytosis events was additionally associated with a decrease in the release of antimicrobial peptides contributing to the decrease in killing of *C. albicans* cells. This in turn lead to an increase in the number of resistant *C. albicans* cells reaching levels that were well-beyond those observed for paralytic monocytes (see Figure 10). Comparing the hypo-inflammatory condition with PMN deficiency, we found that diffusion coefficients around *D_G_* = 100 μm^2^/min resembled the outcome of moderate to severe neutropenia.

The bottom-up modeling approach presented here may be extended in various ways. For example, the implementation of a hybrid ABM could be envisaged where molecular interactions, e.g., as mediated by antimicrobial peptides, are not simulated in a rule-based fashion but in an explicit way by a molecular diffusion solver. Other directions of future research include (i) focusing on conditions of immune dysregulation, (ii) comparing the impact of different pathogens, and (iii) including other types of innate immune cells. Furthermore, it is conceivable to combine modeling approaches with microscopy experiments of infection scenarios *in vitro* in an image-based systems biology approach (Mech et al., 2014; Figge and Murphy, 2015; Medyukhina et al., 2015). First steps into this direction have recently been made, e.g., by establishing algorithms for the automated image analysis of phagocytosis assays (Mech et al., 2011; Kraibooj et al., 2014) and for the automated tracking and classification of PMN from time-lapse microscopy (Mokhtari et al., 2013; Brandes et al., 2015) that was applied in the context of comparing *C. albicans* and *C. glabrata* infection (Duggan et al., 2015a). In the future, we expect that a systems medicine approach exploiting the predictive power of virtual infection models will play an important role in the context of infectious disease diagnosis.

## Author contributions

TL, ST, MTF: Conception and design of the investigation and work. MTF: Contribution of materials and computational resources. TL, ST, JP, MTF: Data processing, implementation and application of the computational algorithm. TL, ST, JP, KH, OK, MTF: Evaluation and analysis of the results. TL, ST, JP, KH, OK, MTF: Drafting the manuscript and revising it critically for important intellectual content and final approval of the version to be published. TL, ST, JP, KH, OK, MTF: Agreement to be accountable for all aspects of the work in ensuring that questions related to the accuracy or integrity of any part of the work are appropriately investigated and resolved.

## Funding

This work was financially supported by the Deutsche Forschungsgemeinschaft (DFG) through the excellence graduate school Jena School for Microbial Communication (JSMC) and the CRC/TR124 FungiNet (project B4 to MTF and project C3 to OK).

### Conflict of interest statement

The authors declare that the research was conducted in the absence of any commercial or financial relationships that could be construed as a potential conflict of interest.
