# Supplementary material for: Bottom-up modeling approach for the quantitative estimation of parameters in pathogen-host interactions
Source: Front Microbiol. 2015 Jun 19;6:608. doi: 10.3389/fmicb.2015.00608 (PMC4473060; doi:10.3389/fmicb.2015.00608)
Supplement: Supplementary file 1 [file DataSheet1.PDF]

Supplementary Material: Bottom-up modeling approach for the quantitative estimation of parameters in pathogen-host interactions

Teresa Lehnert [1,2,+], Sandra Timme [1,2+], Johannes Pollmächer [1,2], Kerstin Hünniger [3], Oliver Kurzai [3,2] and Marc Thilo Figge [1,2*]

[1] *Applied Systems Biology, Leibniz Institute for Natural Product Research and Infection Biology Hans-Knöll-Institute (HKI), Jena, Germany*
[2] *Faculty of Biology and Pharmacy, Friedrich Schiller University Jena, Jena, Germany*
[3] *Fungal Septomics, Septomics Research Center, Friedrich Schiller University and Leibniz Institute for Natural Product Research and Infection Biology Hans-Knöll-Institute (HKI), Jena, Germany*
[+] *Authors contributed equally*

Correspondence*:
Marc Thilo Figge
Applied Systems Biology, Leibniz Institute for Natural Product Research and Infection Biology - Hans Knöll Institute (HKI), Adolf-Reichwein-Straße 23, Jena, 07745, Germany, thilo.figge@hki-jena.de

1 SUPPLEMENTARY DATA

Supplementary Video 1. Typical time evolution of the virtual infection scenario in the spatial ABM. The three-dimensional environment corresponds to 1 μl of blood containing 500 monocytes, 5000 PMN and 1000 *C. albicans* cells. The cells are represented as spherical agents that migrate depending on their respective diffusion coefficients and interact depending on rules as depicted in Figure 3 (B). *C. albicans* cells occur in the four states (i) alive and non-resistant (green), (ii) dead and non-resistant (red), (iii) alive and resistant (yellow) and (iv) dead and resistant (gray). Furthermore, granulocytes (polymorphonuclear neutrophils, PMN) and monocytes are shown in blue and orange, respectively. The video comprises the immune response during four hours post infection. In the beginning of the video, most *C. albicans* cells are non-phagocytosed and are frequently found to be alive and non-resistant (green). Towards the end of the video, non-phagocytosed *C. albicans* cells are mostly resistant (*i.e.*, yellow and gray) while most of *C. albicans* cells have been phagocytosed.

2 SUPPLEMENTARY INFORMATION

2.1 ANNEALING FUNCTION τ

The function $\tau(f)$ determines the acceptance of worse parameters during the applied fitting procedure Metropolis Monte Carlo based on Simulated Annealing. This function of fitting steps f is defined in form of the Hill equation, *i.e.*

$$\tau(f) = \tau_0 + \frac{f^n}{(P^n + f^n)} (\tau_\infty - \tau_0) , \quad (1)$$

that ranges between τ_0 and τ_∞ (see Supplementary Table 1 for applied values) and is characterized by the coefficient $n = 3$ describing the slope of the function as well as the point of inflection $P = 0.5\, f_{total}$ (with f_{total} the total number of fitting steps). We determined the value for n and P by manual adjustment.

3 SUPPLEMENTARY TABLES AND FIGURES

Supplementary Table 1. Score values ϵ_c for combined units c.

combined unit c	score value ϵ_c
C_A	45
C_K	20
C_E	70
C_M	200
C_G	30

Manually adjusted score values ϵ_c of the combined units c that are used for comparison with experimental data from whole-blood infection assay. All states of the PI-SBM that represent alive (killed) *C. albicans* cells are summarized to the combined unit C_A (C_K). States of *C. albicans* cells in extracellular space, in monocytes or in PMN are represented by the combined units C_E, C_M or C_G, respectively.

Supplementary Table 2. Individual start conditions of the fitting algorithm.

$C_{AE}(0)$	$M_{0,0}(0)$	$G_{0,0}(0)$	number of runs	number of fitting steps	τ
100	50	500	100	3 000	[0.05,5.0]
1 000	500	5 000	50	1 000	[5.0;30.0]
10 000	5 000	50 000	20	250	[5.0;80.0]
100 000	50 000	500 000	10	50	[50.0;80.0]

Start conditions for the parameter fitting algorithm. The number of individuals of alive *C. albicans* cells in extracellular space ($C_{AE}(0)$), monocytes ($M_{0,0}(0)$) and PMN ($G_{0,0}(0)$) at time $t = 0$ min was stepwise increased by keeping their ratio constant. For each step, the number of runs with corresponding number of fitting steps per run and the range of τ was adjusted.

Supplementary Table 3. Transition rates for PI-SBM.

	rate $[min^{-1}]$	standard deviation $[min^{-1}]$	standard deviation $[\%]$
ϕ_G	$2.83 \cdot 10^{-2}$	$0.49 \cdot 10^{-3}$	1.7
ϕ_{G*}	$4.75 \cdot 10^{-2}$	$3.86 \cdot 10^{-3}$	8.13
ϕ_M	$1.25 \cdot 10^{-2}$	$0.74 \cdot 10^{-3}$	5.9
κ_M	$3.59 \cdot 10^{-2}$	$3.74 \cdot 10^{-3}$	10.4
κ_G	$4.69 \cdot 10^{-2}$	$4.25 \cdot 10^{-3}$	9.1
ρ	$0.45 \cdot 10^{-2}$	$0.19 \cdot 10^{-3}$	4.2
γ	$1.93 \cdot 10^{-2}$	$2.76 \cdot 10^{-3}$	14.3
$\bar{\kappa}_{EK}$	$26.07 \cdot 10^{-2}$	$22.65 \cdot 10^{-3}$	8.7

The transition rates of the P-SBM are given by the phagocytosis rate ϕ_G of PMN that phagocytose for their first time, the phagocytosis rate $\phi_{G^\star}$ of PMN that phagocytose for at least the second time, the phagocytosis rate ϕ_M of monocytes, the intracellular killing rate κ_M of monocytes, the intracellular killing rate κ_G of PMN, the resistance rate ρ and the rates that determine the extracellular killing κ_{EK} and γ.

Supplementary Table 4. Transition rates for P-SBM.

	rate $[min^{-1}]$	standard deviation $[min^{-1}]$	standard deviation $[\%]$
ϕ_G	$2.69 \cdot 10^{-2}$	$0.33 \cdot 10^{-3}$	1.24
$\phi_{G^\star}$	$3.58 \cdot 10^{-2}$	$1.88 \cdot 10^{-3}$	5.24
ϕ_M	$0.63 \cdot 10^{-2}$	$0.33 \cdot 10^{-3}$	5.25
κ_M	$6.08 \cdot 10^{-2}$	$4.04 \cdot 10^{-3}$	6.64
κ_G	$4.26 \cdot 10^{-2}$	$2.03 \cdot 10^{-3}$	4.76
ρ	$0.41 \cdot 10^{-2}$	$0.13 \cdot 10^{-3}$	3.25
γ	$3.16 \cdot 10^{-2}$	$2.15 \cdot 10^{-3}$	6.8
$\kappa_{\bar{E}K}$	$29.13 \cdot 10^{-2}$	$14.35 \cdot 10^{-3}$	4.93

The transition rates of the PI-SBM are given by the phagocytosis rate ϕ_G of PMN that phagocytose for their first time, the phagocytosis rate $\phi_{G^\star}$ of PMN that phagocytose for at least the second time, the phagocytosis rate ϕ_M of monocytes, the intracellular killing rate κ_M of monocytes, the intracellular killing rate κ_G of PMN, the resistance rate ρ and the rates that determine the extracellular killing κ_{EK} and γ.

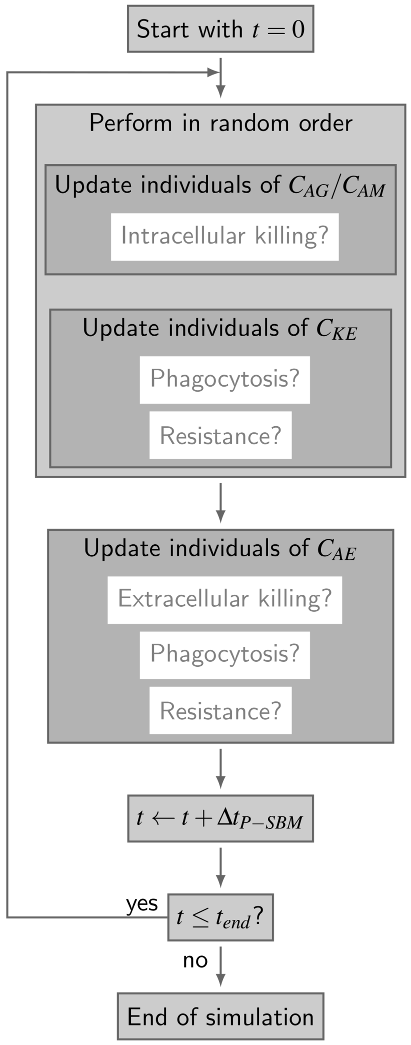

Supplementary Figure 1. Flow-chart of the non-spatial P-SBM simulation algorithm. In each time-step Δt_{P-SBM}, all individuals of *C. albicans* cell states are tested for possible state transitions. Individuals of alive *C. albicans* cells in monocytes (C_{AM}) or PMN (C_{AG}) are tested for intracellular killing. Individuals of the state killed-and-extracellular *C. albicans* cells (C_{KE}) are tested for phagocytosis and for becoming resistant. The individuals of the state alive-and-extracellular *C. albicans* cells (C_{AE}) are tested for phagocytosis, for extracellular killing and for becoming resistant.

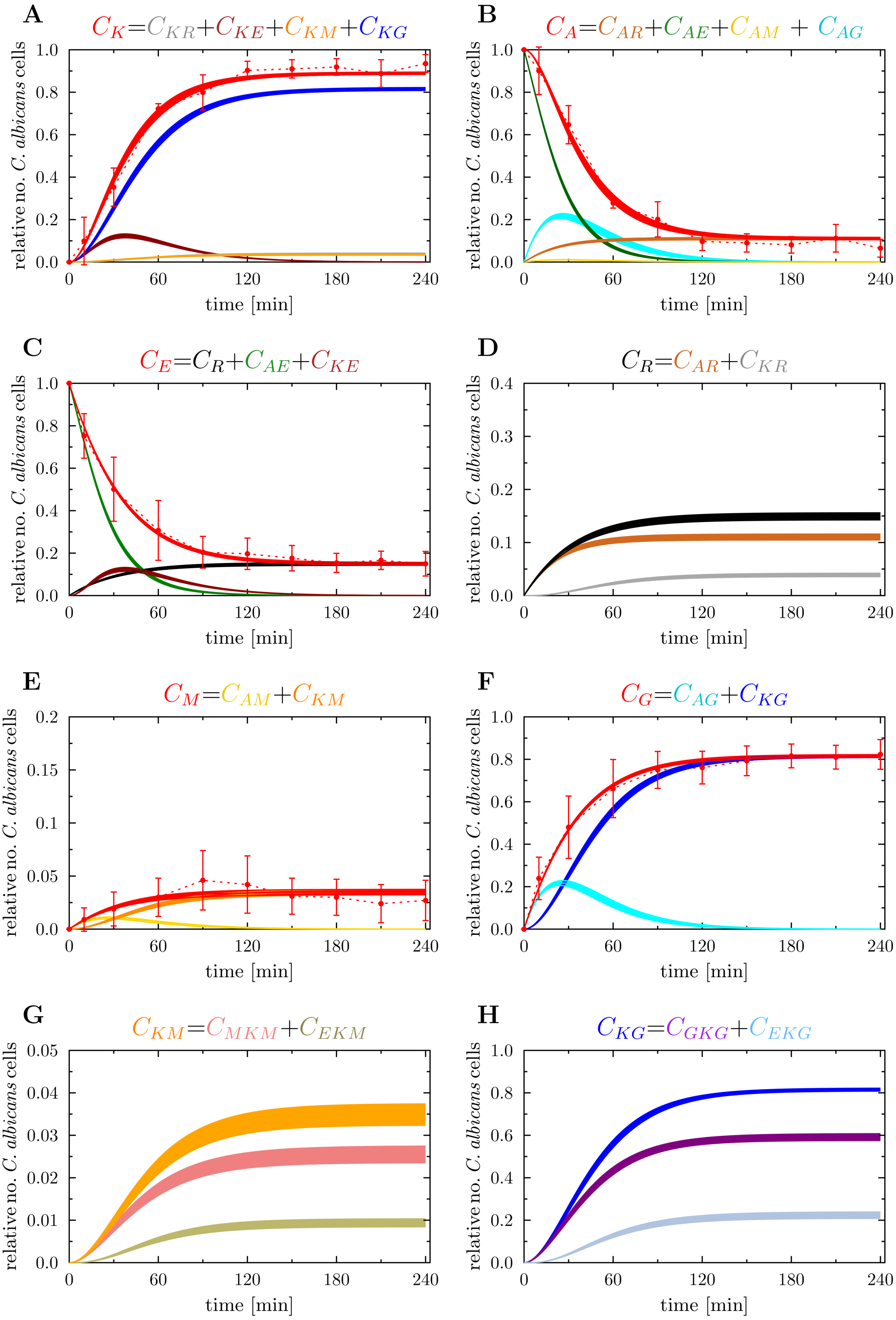
A
C_K=C_{KR}+C_{KE}+C_{KM}+C_{KG}
B
C_A=C_{AR}+C_{AE}+C_{AM} + C_{AG}
C
C_E=C_R+C_{AE}+C_{KE}
D
C_R=C_{AR}+C_{KR}
E
C_M=C_{AM}+C_{KM}
F
C_G=C_{AG}+C_{KG}
G
C_{KM}=C_{MKM}+C_{EKM}
H
C_{KG}=C_{GKG}+C_{EKG}
relative no. *C. albicans* cells
time [min]
0 60 120 180 240

Supplementary Figure 2. Kinetics of PI-SBM simulations (red solid lines) for the minimal least squares error (LSE) relative to the experimental data from whole-blood infection assays (red dotted lines as guide for the eye). The error bars correspond to the standard deviations of five independent experiments. The thickness of the solid lines represents the standard deviation of the simulation results as obtained from 50 simulations for normally distributed transition rates as given in Supplementary Table 2. Colored symbols refer to different *C. albicans* states, where their time courses are indicated by continuous lines with the same color. (A) Time-dependent relative number of killed *C. albicans* cells (C_K) that were experimentally measured by survival plates. The experimental results were compared with the combination of simulated data representing all killed *C. albicans* of the model, *i.e.* extracellularly killed *C. albicans* (C_{KE}), killed resistant *C. albicans* (C_{KR}), killed *C. albicans* that are in monocytes (C_{KM}) or PMN (C_{KG}). (B) Alive *C. albicans* (C_A) that were measured by survival plates and simulated by the combination of alive *C. albicans* that are in extracellular space (C_{AE}), in monocytes (C_{AM}), in PMN (C_{AG}) or became resistant against phagocytosis (C_{AR}). (C) Time course of *C. albicans* cells that are in extracellular space of blood (C_E). Experimental data was obtained by FACS analysis and simulated data is represented by the combination of *C. albicans* cells that are extracellular alive (C_{AE}), extracellularly killed (C_{KE}) and resistant against phagocytosis (C_R). (D) The simulated resistant *C. albicans* (C_R) are the sum of alive and dead resistant *C. albicans* cells at each time point of the simulation time. (E) Time course of *C. albicans* cells that were phagocytosed by monocytes (C_M). This is defined as sum of alive and killed *C. albicans* cells in monocytes, *i.e.* C_{AM} and C_{KM}, respectively. The corresponding experimental data was obtained by FACS analysis. (F) Relative number of *C. albicans* cells in PMN (C_G) during the whole-blood infection, where internalized *C. albicans* cells can be alive (C_{AG}) or dead (C_{KG}). (G) Simulation result of killed *C. albicans* cells within monocytes (C_{KM}), that is defined as the sum of internalized *C. albicans* that were intracellularly killed(C_{MKM}) and those who were extracellularly killed (C_{EKM}). (H) Simulated time course of killed *C. albicans* cells in PMN (C_{KG}), that is composed of intracellularly killed *C. albicans* cells (C_{GKG}) and extracellularly killed *C. albicans* cells (C_{EKG}) in PMN.

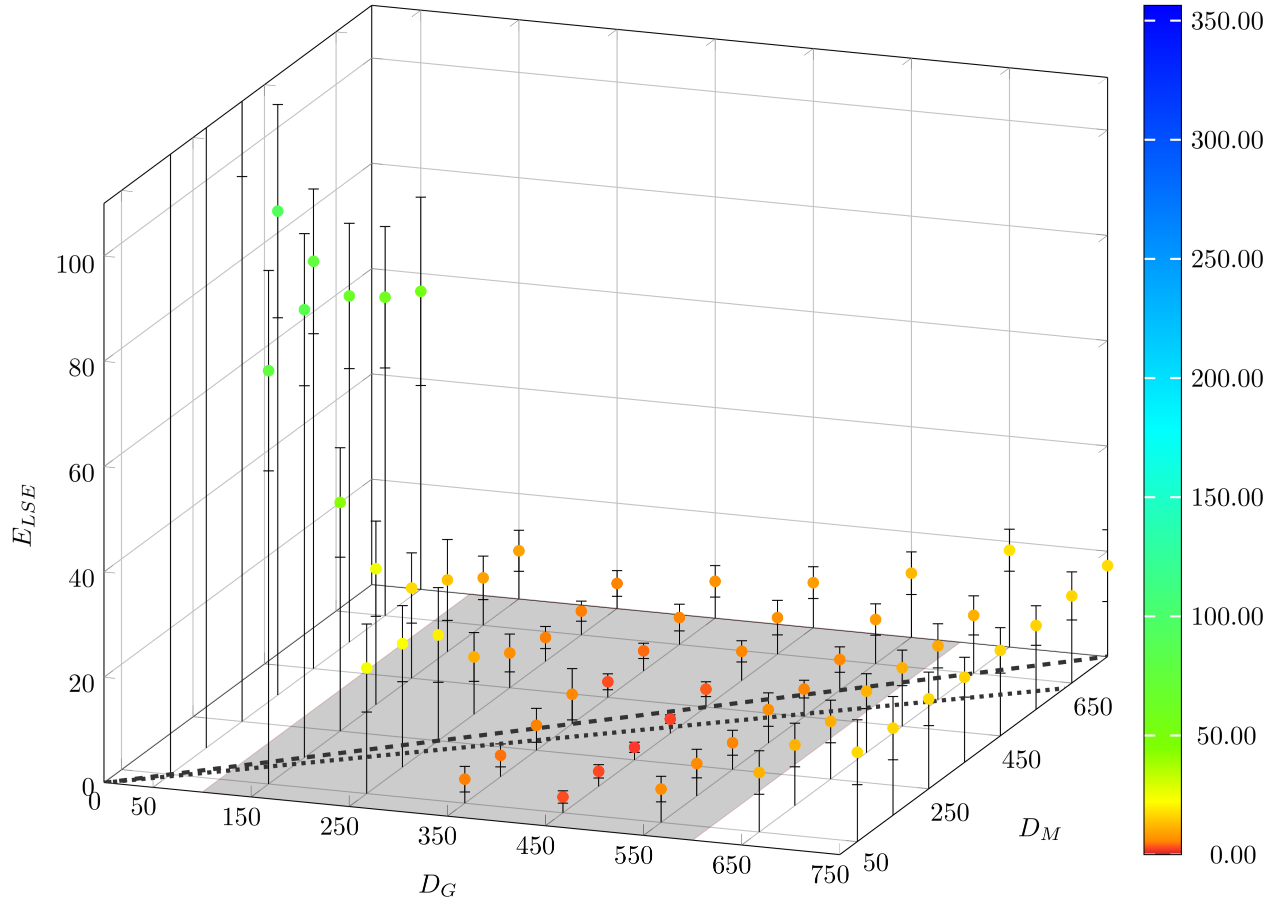

Supplementary Figure 3. Result of ABM parameter estimation by *adaptive regular grid search* applied to the small ABM representing $1/5\,\mu l$ blood. The space of diffusion coefficients of monocytes (D_M) and PMN (D_G) was scanned at the grid points in the pre-defined area. At each grid point the mean value of the least squares error (LSE) and the standard deviation over 30 simulations are shown. The gray region represents the rectangle that contains all pairs of diffusion coefficients for which the LSE values were found to be minimal from separately varying each diffusion coefficient. The dashed line corresponds to $D_M = D_G$ and the dotted line corresponds to diffusion coefficients that follow the Stokes-Einstein equation for monocytes and PMN, *i.e.* $D_G/D_M = d_M/d_G$ with monocyte diameter $d_M = 16\,\mu m$ and PMN diameter $d_G = 13.5\,\mu m$.

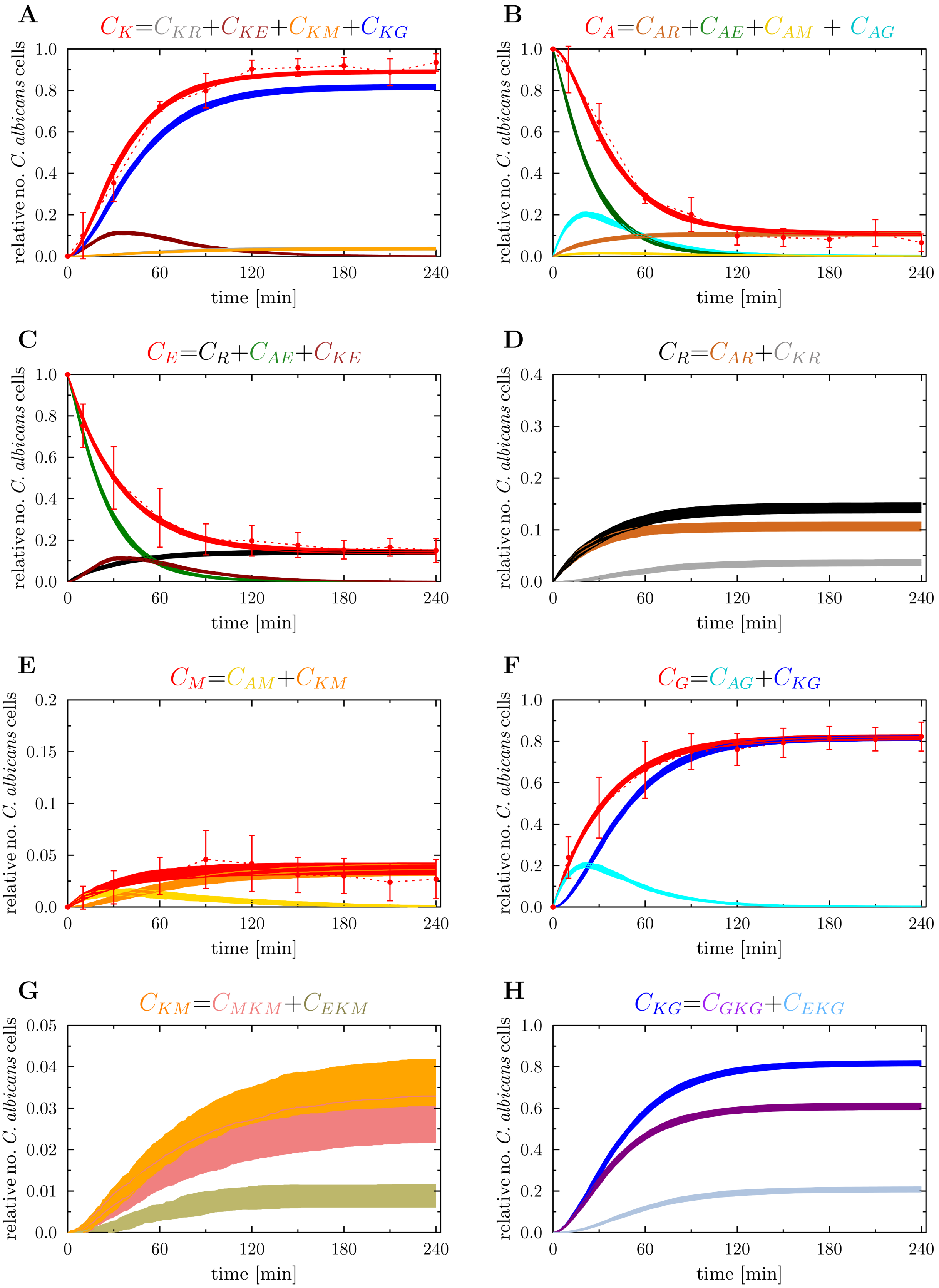
A
$C_K = C_{KR} + C_{KE} + C_{KM} + C_{KG}$
B
$C_A = C_{AR} + C_{AE} + C_{AM} + C_{AG}$
C
$C_E = C_R + C_{AE} + C_{KE}$
D
$C_R = C_{AR} + C_{KR}$
E
$C_M = C_{AM} + C_{KM}$
F
$C_G = C_{AG} + C_{KG}$
G
$C_{KM} = C_{MKM} + C_{EKM}$
H
$C_{KG} = C_{GKG} + C_{EKG}$
relative no. *C. albicans* cells
time [min]
0
60
120
180
240

Supplementary Figure 4. Kinetics of ABM simulations with $(D_G^{min}, D_M^{min}) = (425, 275)\, \mu m^2/min$ (red solid lines) for the minimal least squares error (LSE) relative to the experimental data from whole-blood infection assays (red dotted lines as guide for the eye). The error bars correspond to the standard deviations of five independent experiments. The thickness of the solid lines represents the standard deviation of the simulation results as obtained from 30 simulations. Colored symbols refer to different *C. albicans* states, where their time courses are indicated by continuous lines with the same color. (A) Time-dependent relative number of killed *C. albicans* cells (C_K) that were experimentally measured by survival plates. The experimental results were compared with the combination of simulated data representing all killed *C. albicans* of the model, *i.e.* extracellularly killed *C. albicans* (C_{KE}), killed resistant *C. albicans* (C_{KR}), killed *C. albicans* that are in monocytes (C_{KM}) or PMN (C_{KG}). (B) Alive *C. albicans* (C_A) that were measured by survival plates and simulated by the combination of alive *C. albicans* that are in extracellular space (C_{AE}), in monocytes (C_{AM}), in PMN (C_{AG}) or became resistant against phagocytosis (C_{AR}). (C) Time course of *C. albicans* cells that are in extracellular space of blood (C_E). Experimental data was obtained by FACS analysis and simulated data is represented by the combination of *C. albicans* cells that are extracellular alive (C_{AE}), extracellularly killed (C_{KE}) and resistant against phagocytosis (C_R). (D) The simulated resistant *C. albicans* (C_R) are the sum of alive and dead resistant *C. albicans* cells at each time point of the simulation time. (E) Time course of *C. albicans* cells that were phagocytosed by monocytes (C_M). This is defined as sum of alive and killed *C. albicans* cells in monocytes, *i.e.* C_{AM} and C_{KM}, respectively. The corresponding experimental data was obtained by FACS analysis. (F) Relative number of *C. albicans* cells in PMN (C_G) during the whole-blood infection, where internalized *C. albicans* cells can be alive (C_{AG}) or dead (C_{KG}). (G) Simulation result of killed *C. albicans* cells within monocytes (C_{KM}), that is defined as the sum of internalized *C. albicans* that were intracellularly killed(C_{MKM}) and those who were extracellularly killed (C_{EKM}). (H) Simulated time course of killed *C. albicans* cells in PMN (C_{KG}), that is composed of intracellularly killed *C. albicans* cells (C_{GKG}) and extracellularly killed *C. albicans* cells (C_{EKG}) in PMN.
